# Desorption of Lipases Immobilized on Octyl-Agarose Beads and Coated with Ionic Polymers after Thermal Inactivation. Stronger Adsorption of Polymers/Unfolded Protein Composites

**DOI:** 10.3390/molecules22010091

**Published:** 2017-01-05

**Authors:** Jose J. Virgen-Ortíz, Sara G. Pedrero, Laura Fernandez-Lopez, Nerea Lopez-Carrobles, Beatriz C. Gorines, Cristina Otero, Roberto Fernandez-Lafuente

**Affiliations:** 1 CONACYT—Centro de Investigación en Alimentación y Desarrollo, A.C. (CIAD)—Centro de Innovación y Desarrollo Agroalimentario de Michoacán, A.C. (CIDAM), Km. 8 Antigua Carretera a Pátzcuaro s/n, C.P. 58341 Morelia, Michoacán, Mexico; juanvirgen@hotmail.com; 2 Departamento de Biocatálisis, Instituto de Catálisis-CSIC, C/Marie Curie 2, Campus UAM-CSIC, Cantoblanco, 28049 Madrid, Spain; saramg04@ucm.es (S.G.P.); laura_valde95@hotmail.com (L.F.-L.); nelopez@ucm.es (N.L.-C.); beatrcha@ucm.es (B.C.G.); cotero@icp.csic.es (C.O.)

**Keywords:** enzyme physical crosslinking with polymers, octyl-agarose, lipase immobilization, enzyme desorption, support reuse, enzyme inactivation

## Abstract

Lipases from *Candida antarctica* (isoform B) and *Rhizomucor miehei* (CALB and RML) have been immobilized on octyl-agarose (OC) and further coated with polyethylenimine (PEI) and dextran sulfate (DS). The enzymes just immobilized on OC supports could be easily released from the support using 2% SDS at pH 7, both intact or after thermal inactivation (in fact, after inactivation most enzyme molecules were already desorbed). The coating with PEI and DS greatly reduced the enzyme release during thermal inactivation and improved enzyme stability. However, using OC-CALB/RML-PEI-DS, the full release of the immobilized enzyme to reuse the support required more drastic conditions: a pH value of 3, a buffer concentration over 2 M, and temperatures above 45 °C. However, even these conditions were not able to fully release the thermally inactivated enzyme molecules from the support, being necessary to increase the buffer concentration to 4 M sodium phosphate and decrease the pH to 2.5. The formation of unfolded protein/polymers composites seems to be responsible for this strong interaction between the octyl and some anionic groups of OC supports. The support could be reused five cycles using these conditions with similar loading capacity of the support and stability of the immobilized enzyme.

## 1. Introduction

Enzyme immobilization is a requirement in the design of most biocatalysts to solve the problem of enzyme solubility [1]. Considering this, many authors have tried to develop strategies that allow improving other enzyme properties during this step. This way, stability, activity, resistance to inhibitors, selectivity, specificity or even purity may be improved if a proper immobilization protocol is applied [2,3,4,5,6,7,8,9,10,11,12,13,14,15].

Reversible physical immobilization of enzymes has some advantages regarding covalent immobilization: the support may be reused after inactivation, saving costs of support expenses and disposal, especially when it is not biodegradable, the support groups are stable for long time periods even under non-controlled temperatures, and immobilization protocols are very simple [16].

However, these strategies have two main problems. First, there is the risk of enzyme desorption during operation. This produces apparent inactivation of the biocatalyst by loss of active protein and product contamination by the enzyme. Second, stabilization achieved via physical immobilization is usually moderate, because the support remains necessarily physically active (inert supports are preferred to prevent enzyme-support undesired interactions) [17], and usually cannot compete with multipoint covalent attachment [16].

However, there are some examples where physical adsorption is very strong and the enzyme stability is improved significantly. For example, when a multimeric enzyme is immobilized via ion exchange involving all enzyme subunits, and dissociation is no longer possible [18,19]. There is a case where stability using physical adsorption is even higher than those obtained via multipoint covalent attachment [20,21]. This is the case of lipases adsorbed on hydrophobic supports via interfacial activation [22]. Lipases are enzymes that exhibit a peculiar mechanism of action, presenting in homogeneous media the active center isolated from the medium by a polypeptide chain called lid [23,24,25,26,27]. The surroundings of the active center and the internal face of the lid are hydrophobic. This characteristic makes the open form of lipases unstable in homogenous aqueous media. Lipase open forms are strongly adsorbed on drops of apolar substrates (e.g., oil or fats). That way the active center becomes accessible to substrates and the enzyme may act in the interface [28,29,30,31,32,33]. This mechanism of action has permitted to develop strategies for the one step purification, immobilization, hyperactivation and stabilization of lipases. It is based on the use of hydrophobic supports and performing the immobilization at low ionic strength [34,35]. The adsorbed open form is far more stable than the lipases in equilibrium between closed and open forms [36], and these preparations become more thermostable even than lipases submitted to an intense multipoint covalent attachment [20,21].

Lipases are enzymes widely used in biocatalysis due to their high stability, wide specificity coupled to a strict selectivity or specificity in certain cases, ability to catalyze different reactions, etc. [23,37,38,39]. Although lipase immobilization on hydrophobic supports is strong, the lipase may be released from the support (e.g., using detergents), and the support may be reused [34,35]. Unfortunately, enzyme desorption may also occur under drastic conditions (high temperature, presence of organic solvents) [40], and some substrates or products may even facilitate enzyme release (e.g., free fatty acid, monoacetin o monobutyrin) [41]. The coating of the immobilized lipases with PEI and dextran sulfate revealed itself as a powerful tool to prevent enzyme desorption and to improve enzyme stability. This is due, among other reasons, to the fact that it may prevent enzyme desorption via intermolecular physical crosslinking [42,43,44]. This treatment remains only a physical one, and it has been stated as reversible even though desorption of the crosslinked lipases becomes quite difficult and it requires the use of ionic detergents, high ionic strength, low pH value and moderate temperature [43,44].

On the other hand, it has been shown that ionic exchangers may adsorb the inactivated enzyme molecules much more strongly than native ones, because the inactivated enzyme is unfolded, thus maximizing the number of enzyme/support interactions and that way, making enzyme desorption from anion exchangers harder [45].

The use of octyl agarose beads to immobilize lipase is apparently different, as the immobilization is based in a pseudo-affinity between the enzyme and the octyl layer in the surface of the support [22]. Moreover, the enzyme is released to the medium during both thermal and solvent inactivations, making the desorption of the lipase molecules from the support easy [40]. However, it has been reported that this support presents some anionic groups on its surface, making the adsorption of PEI molecules on the support surface possible [46]. This possible interaction between PEI molecules and the anionic groups of octyl agarose beads may complicate the regeneration of the support (for reuse the support, it is convenient to have a fully clean one) after enzyme inactivation. First, the PEI may become strongly adsorbed on the support during heating by an increase in its mobility. Second, if some molecules of lipase are unfolded in contact with the PEI, they can also maximize the interaction with the PEI, as it is the case of immobilized PEI molecules and proteins [46]. This may permit some inactivated and unfolded enzyme molecules to become strongly adsorbed onto octyl agarose via a mixed ion exchange/hydrophobic interaction with the octyl and the PEI groups. That way, the recovery of an octyl-agarose support completely free of enzyme, once inactivation of lipases occurs, may become far harder in the case of inactivated lipases coated with PEI and dextran sulfate than in the case of intact immobilized lipases.

In this paper, we have analyzed the possibility that this strong adsorption of PEI-unfolded protein composite may really exist, and, if it does occur, to develop protocols that permit full desorption of the lipase and the PEI from the octyl agarose beads support. This will be also valid to regenerate the support when the lipase immobilized in octyl support is coated with PEI and used to immobilize a second enzyme over it, as described in [47].

## 2. Results and Discussion

### 2.1. Recovery of Octyl-Agarose from OC-CALB and OC-RML Preparations

**Figure 1:**
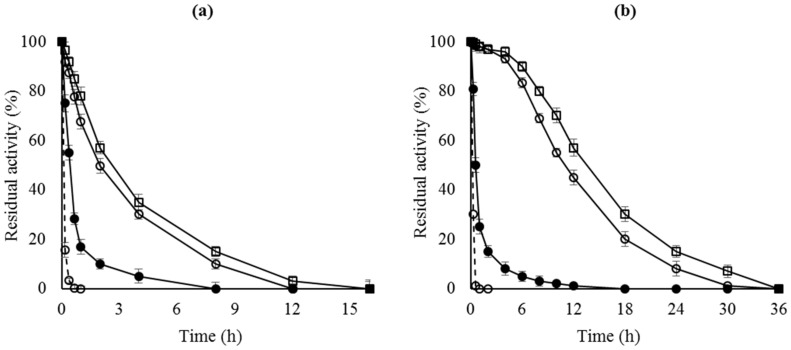
Thermal inactivation courses of different octyl-lipase biocatalysts. (**a**) CALB preparations inactivated at pH 7 and 68 °C; (**b**) RML preparations inactivated at pH 7 and 45 °C. Dashed line, open circles: lipase free; close circles: OC-lipase; triangles: OC-lipase-PEI; squares: OC-lipase-PEI-DS.

Figure 1 shows that the enzyme stability of the OC preparations was higher than that of the free enzyme. When analyzing the enzyme remaining in the support after inactivation, most of the enzyme had been released to the medium (results not shown), as previously reported [40]. Incubation of the enzyme with 2% Triton X-100 (*v*/*v*) or SDS (*w*/*v*) in 25 mM sodium phosphate at pH 7 is enough to recover a fully clean support both, before and after enzyme inactivation.

### 2.2. Recovery of Octyl-Agarose from OC-CALB-PEI-DS and OC-RML-PEI-DS Preparations

Figure 1 shows that enzyme stability was greatly improved after the polymer coating of the OC immobilized enzymes, mainly in the case of RML. This was attributed to the intermolecular physical crosslinking of the immobilized lipases. Now enzyme desorption during thermal inactivation is significantly reduced [43,44].

**Figure 2:**
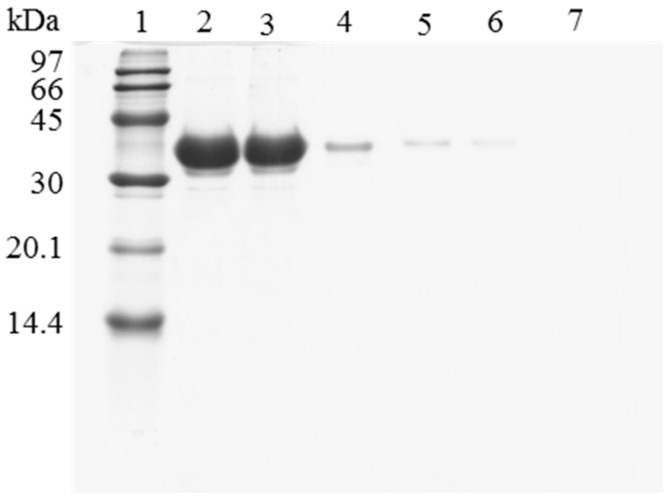
SDS-PAGE analysis of OC-CALB biocatalysts coating with polyethylenimine and dextran sulfate after desorption of the lipase without inactivation process. The biocatalyst was submitted to the processes described in the Experimental section. Gel shows the enzyme that remains bound to the support after desorption. Unless otherwise indicated, all the desorption experiments were performed at 25 °C. Lane 1: molecular weight marker. Lane 2: OC-CALB derivative. Lane 3: OC-CALB-PEI-DS derivative. Lane 4: after desorption with 2% SDS (pH 7). Lane 5: desorption with 2% SDS in 2 M sodium phosphate (pH 7). Lane 6: desorption with 2% SDS in 2 M sodium phosphate (pH 3). Lane 7: desorption with 2% SDS in 2 M sodium phosphate (pH 3) at 45 °C.

However, when trying to reuse the support, it was found that the enzyme release was greatly hindered compared to using the untreated OC lipase preparation. Figure 2 shows the SDS-PAGE analysis of the protein that remained attached to the support after being submitted to different washing protocols. The results show how the recovery of a fully clean support using OC-CALB-PEI-DS is more difficult than when using OC-CALB. Now, using 2% SDS at pH 7 and 25 °C, (conditions that permitted the full release of the untreated enzyme) a large amount of enzyme molecules remained attached to the support using the polymer coated biocatalyst. This should be related to the physical intermolecular crosslinking of the enzymes. After enzyme coating with the polymers, it is necessary to simultaneously desorb all the enzyme molecules that are physically crosslinked, this is far more complex that to desorb an individual lipase molecule. This is a new evidence that the main effect of coating the immobilized lipases with ionic polymers is the prevention of enzyme desorption during inactivation [43,44]. However, when the aim is to desorb the immobilized enzyme molecules, this may become a problem. To reach the objective of making enzyme desorption easier, ionic strength was increased and the pH was lowered to weaken the enzyme-PEI interactions. Figure 2 shows that the consecutive use of a higher buffer concentration, a lower pH value (to decrease ionic interactions), and the increase of the Temperature to 45 °C were necessary to get a full desorption of the enzyme immobilized in the support. This demonstrated the high efficiency of the system to prevent enzyme desorption, as it was intended [42,44].

**Figure 3:**
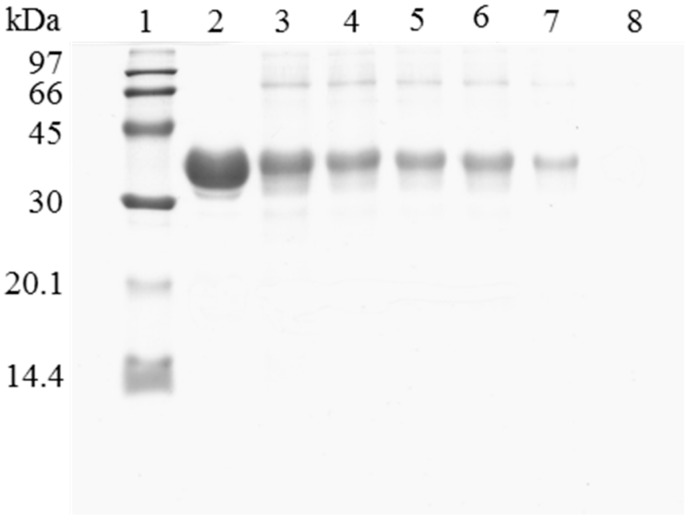
SDS-PAGE analysis of CALB biocatalysts coating with PEI and DS after desorption of the inactivated lipase. The biocatalyst was submitted to the processes described in the Experimental section. Gel shows the protein that remains bound to the support after desorption. Unless otherwise indicated, all the desorption experiments were performed at 25 °C. Lane 1: molecular weight marker. Lane 2: OC-CALB-PEI-DS derivative. Lane 3: after thermal inactivation. Lane 4: desorption with 2% SDS (pH 7). Lane 5: desorption with 2% SDS in 2 M sodium phosphate (pH 7). Lane 6: desorption with 2% SDS in 2 M sodium phosphate (pH 3). Lane 7: desorption with 2% SDS in 2 M sodium phosphate (pH 3) at 45 °C. Lane 8: desorption with 2% SDS in 4 M sodium phosphate (pH 2.5) at 45 °C.

**Figure 4:**
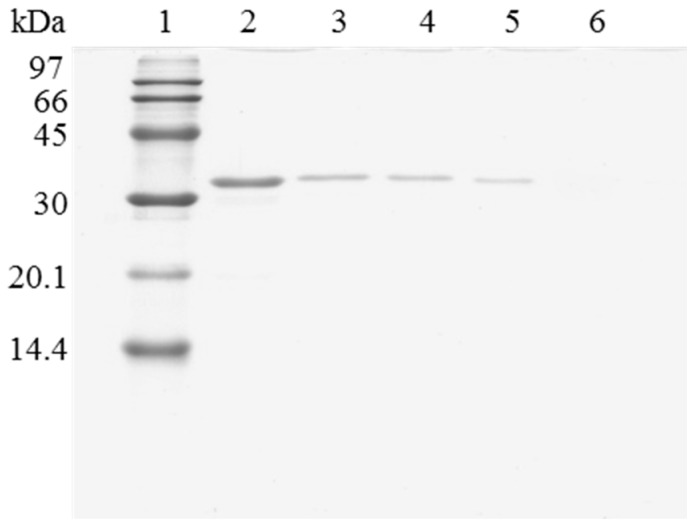
SDS-PAGE analysis of OC-RML biocatalysts coating with PEI and DS after desorption of the lipase without inactivation process. The biocatalyst was submitted to the processes described in the Experimental section. Gel shows the enzyme that remains bound to the support after desorption. Unless otherwise indicated, all the desorption experiments were performed at 25 °C. Lane 1: molecular weight marker. Lane 2: OC-RML-PEI-DS derivative. Lane 3: after desorption with 2% SDS (pH 7). Lane 4: desorption with 2% SDS in 2 M sodium phosphate (pH 7). Lane 5: desorption with 2% SDS in 2 M sodium phosphate (pH 3). Lane 6: desorption with 2% SDS in 2 M sodium phosphate (pH 3) at 45 °C.

**Figure 5:**
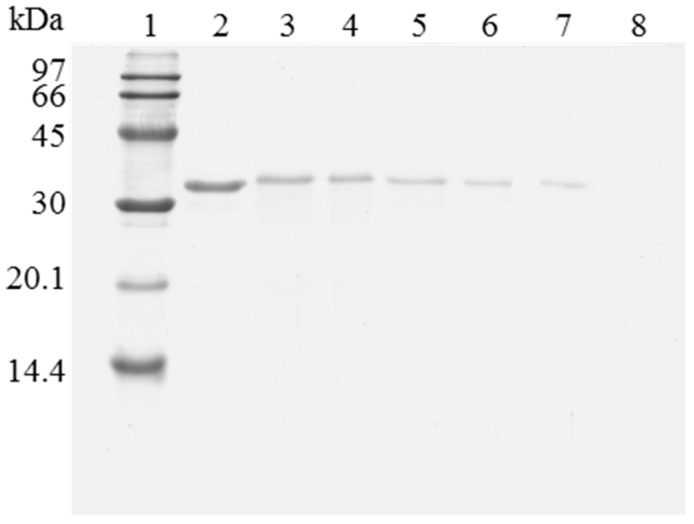
SDS-PAGE analysis of OC-RML biocatalysts coating with PEI and DS after desorption of the inactivated lipase. The biocatalyst was submitted to the processes described in the Experimental section. Gel shows the protein that remains bound to the support after desorption. Unless otherwise indicated, all the desorption experiments were performed at 25 °C. Lane 1: molecular weight marker. Lane 2: OC-RML-PEI-DS derivative. Lane 3: after thermal inactivation. Lane 4: desorption with 2% SDS (pH 7). Lane 5: desorption with 2% SDS in 2 M sodium phosphate (pH 7). Lane 6: desorption with 2% SDS in 2 M sodium phosphate (pH 3). Lane 7: desorption with 2% SDS in 2 M sodium phosphate (pH 3) at 45 °C. Lane 8: desorption with 2% SDS in 4 M sodium phosphate (pH 2.5) at 45 °C.

When the immobilized and polymer coated biocatalyst was submitted to inactivation, a significant amount of enzyme remained immobilized (Figure 3). To have a clean support, it is necessary to release this enzyme from the support. However, in this case, the use of 2% SDS in 2 M sodium phosphate at pH 3 and 45 °C was not enough for fully release the enzyme from the support. Only increasing the ionic strength to 4 M sodium phosphate, lowering the pH at 2.5 and working at 45 °C, a fully clean support could be obtained. Similar results were obtained using OC-RML-PEI-DS (Figure 4 and Figure 5); the enzyme was partially desorbed during thermal inactivation (in a much smaller proportion than the uncoated preparations), but the enzyme molecules that remained attached to the support remained thus under conditions where the non-inactivated enzyme was fully desorbed.

Thus, full desorption of lipase molecules adsorbed on octyl agarose beads becomes much harder after their coating with ionic polymers, and become much harder after inactivation. This last result agreed with the results using supports coated with PEI (in fact desorption conditions after enzyme inactivation become similar) and suggested that some of the enzyme molecules may become fully unfold to maximize the unfolded enzyme-polymer interactions (perhaps forming composites involving several enzymes and polymer molecules) when they are inactivated in the presence of ion polymers, making a strong mixed adsorption on the octyl agarose beads (in the acyl chains and also in the anionic groups) [45]. This result may be extrapolated to any other hydrophobic support that has some additional ionic groups, even with a low density that may be unable to directly adsorb proteins via ion exchange. Ionic polymers have a high density of ion groups and a large size, enabling adsorption of the polymer even if the ion groups in the support are very far from each other. And that is more likely if a physical aggregated of several enzymes and polymer molecules is formed.

We also followed the amount of PEI that remained attached to the support under the different treatments using TNBS to titrate the primary amino groups. This will also color the primary amino groups of the protein, but PEI is the main contributor to the final absorbance (Table 1). Table 1 shows that after thermal inactivation, both enzymes lost a significant proportion of PEI (1/3) in both cases. The incubation of the composite in 2% SDS at pH 7 and 25 °C is not able to reduce the PEI more than thermal inactivation, and when the previously inactivated preparations are analyzed, no further decrease of absorbance was detected, suggesting that the weakest adsorbed molecules were desorbed at either, high Temperature or presence of SDS, but the remaining ones were very strongly adsorbed. The use of progressively more drastic desorption conditions permitted to further release more PEI and protein molecules. Using the non-inactivated preparations, the use of 2% SDS in 2 M sodium phosphate at pH 3 and 45 °C was enough to fully release the PEI and the protein, as no absorbance could be detected after treating the washed support with TNBS (that is, neither protein nor PEI remained attached to the support).

Using the inactivated biocatalyst, a significant percentage of PEI remained attached to the support after incubation under these conditions. However, using the support just coated with PEI (PEI can adsorb on the OC support surface) [46], all PEI molecules were released in 2 M sodium phosphate at pH 3 and 45 °C [46]. Thus, if seems that the simultaneous presence of both, PEI and inactivated protein is necessary to have this stronger adsorption of PEI and protein adsorption on octyl agarose beads. As in the case of supports activated with PEI [45], this may be a consequence of the unfolding and maximizes ion exchange of the enzyme and the polymer, which in this case reinforces the interaction of the PEI and the protein with the octyl-agarose surface combining ionic and hydrophobic interactions. The use of a lower pH value and a higher ionic strength permitted to have a fully clean support even after enzyme thermal inactivation. That is, to fully clean the octyl support may be very difficult, but it is still possible and therefore, the support may be reused to immobilized fresh enzyme.

### 2.3. Reuse of Octyl-Agarose to Immobilize Lipase

**Figure 6:**
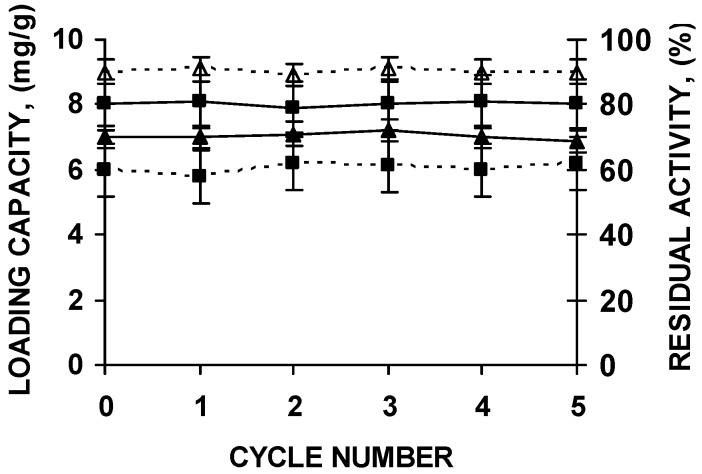
Stability of the biocatalyst and loading capacity of octyl agarose beads during successive reuses of the support after washing under the conditions described in the text. CALB (squares) and RML (triangles) were immobilized in octyl agarose and coated with the polymers as described in the Materials and Methods section. Loading capacity of the support is in continuous lines. Residual activity after incubations for 8 h at pH 7 and 66 °C (CALB) or 47 °C (RML) of the PEI and DS coated biocatalyst is represented in dashed lines.

The supports could be submitted to five cycles of enzyme immobilization, polymer coating, thermal inactivation and desorption without any change in support loading capacity or enzyme stability (Figure 6).

## 3. Materials and Methods

### 3.1. Materials

Commercial soluble lipases from *Candida antarctica* (isoform B) and *Rhizomucor miehie* were kindly donated by Novozymes (Alcobendas, Spain). Octyl-agarose CL-4B beads were from GE Healthcare (Uppsala, Sweden). Polyethylenimine (Mw 25,000 Da), dextran sulfate (average Mw 20,000 Da), *p*-nitrophenyl butyrate (*p*-NPB) and 2,4,6-trinitrobenzenesulfonic acid solution (TNBS, 1 M in water) were purchased from Sigma-Aldrich (St. Louis, MO, USA). Bradford protein assay kit and electrophoresis purity reagents were obtained from Bio-Rad (Hercules, CA, USA). Other reagents were of analytical grade.

### 3.2. Assay of Lipase Activity

Activity was determined by measuring the increase in the absorbance at 348 nm produced by the release of *p*-nitrophenol in the hydrolysis of 0.4 mM *p*-NPB in 25 mM sodium phosphate buffer at pH 7 and 25 °C. The coefficient of extinction (ε) of *p*-nitrophenol, under the conditions described, is 5150 M^−1^cm^−1^. The reaction was initiated with the addition of 50–100 µL of soluble enzyme solution or suspension to a 2.55-mL substrate solution. The assay was carried out using a spectrophotometer (V-630, Jasco Spain (Madrid, Spain)) equipped with a thermostatic cell and continuous magnetic stirring. The initial rates of all enzyme reactions were corrected for the rate of spontaneous hydrolysis of *p-*NPB under identical conditions without enzyme solution. One unit of activity (U) was defined as the amount of enzyme that hydrolyzes 1 µmol of *p*-NPB per minute under the conditions described previously. Protein concentration was measured according to Bradford’s method [48] using bovine serum albumin as a standard.

### 3.3. Immobilization of Lipases on Octyl-Agarose

Soluble CALB and RML were immobilized on octyl-agarose beads at low ionic strength using conditions previously described [34,35]. The commercial solution of the lipase was diluted to 0.2 mg/mL in the corresponding volume of 5 mM sodium phosphate buffer (pH 7). Then, octyl-agarose beads were added under continuous stirring at 25 °C. Derivatives were prepared with the maximum enzymatic loading: 8 mg of CALB per gram of octyl-agarose and 7 mg of RML per gram of support [48]. Activity of supernatant and suspension was followed using *p*-NPB assay. After full enzyme immobilization, the suspension was filtered and the derivative was washed several times with distilled water.

### 3.4. Preparation of Coated Lipase-Octyl-Agarose Derivatives

After the immobilization of lipases on octyl-agarose, the derivatives were coated with polyethylenimine (1%, *w*/*v*) and dextran sulfate (1%, *w*/*v*) [43,44]. A sample of 10 g of wet OC-CALB or OC-RML derivatives were added to 100 mL of PEI solution prepared in 50 mM sodium phosphate buffer at pH 7.0. Suspensions were submitted to gentle stirring for 8 h at 25 °C, and then it was filtered and rinsed thoroughly with distilled water. Modification of the OC-lipase-PEI derivative with dextran sulfate was performed as follows: 10 g of wet OC-CALB-PEI or OC-RML-PEI derivative were suspended in 100 mL of 1% (*w*/*v*) DS solution and pH 7 and the suspension was kept under gentle stirring overnight at 25 °C. Finally, the derivative was washed with distilled water, in a glass funnel, to eliminate the free DS and stored refrigerated (4 °C) until use. The activity of the coated enzymes was almost maintained after the treatments, even slightly improved under certain conditions [42,43,44,45].

### 3.5. Thermal Inactivation of Coated Lipase-Octyl-Agarose Derivatives

Thermal inactivation of OC-CALB-PEI-DS or OC-RML-PEI-DS derivative was performed by incubating 1 g of biocatalyst in 10 mL of 50 mM sodium phosphate buffer (pH 7.0) at 66/68 °C or 45/47 °C for CALB or RML, respectively. Periodically, samples were withdrawn, and their residual activities were determined by *p*-NPB assay. Thermal inactivation was stopped when enzyme activity was no detected.

### 3.6. Enzyme Desorption from Octyl-Agarose Support

To investigate the recovery and reusability of the octyl-agarose support after inactivation of coated lipase-octyl agarose derivatives, a sample of 1 g of the inactivated derivative was suspended in 20 mL of the desorption solution and incubated for two hours at the desired temperature. The solutions and temperature of incubation used for the desorption tests were: 2% SDS in 25 mM sodium phosphate buffer at pH 7 and 25 °C, 2% SDS in 2 M sodium phosphate buffer at pH 7 and 25 °C, 2% SDS in 2 M sodium phosphate buffer at pH 3 and 25 °C or 45 °C, and 2% SDS in 4 M sodium phosphate buffer at pH 2.5 and at 45 °C. After desorption treatment, the support was filtered, washed thoroughly with desorption solution at the same temperature (to prevent enzyme readsorption onto the support) and analyzed by SDS-PAGE for residual adsorbed proteins.

### 3.7. Electrophoretic Analysis

SDS-polyacrylamide gel electrophoresis of the proteins was performed on 15% resolving gel with 5% stacking gel according to Laemmli [49]. To analyze the amount of proteins that remains adsorbed to the octyl-agarose, before or after the desorption tests, 100 mg of the derivative were suspended in 1 mL of rupture buffer (2% SDS and 10% mercaptoethanol). The samples were incubated in a boiling water bath for 8 min and a 10-µL aliquot of the supernatant was loaded to the sample well. The samples were run at 80 volts until the lowest marker reached the lower edge of the gel. Gels were stained with Coomassie brilliant blue. A low molecular weight calibration kit for SDS electrophoresis (GE Healthcare, Barcelona, Spain) was used as a molecular weight marker (14.4–97 kDa).

### 3.8. Determination of the Residual Content of Polyethylenimine from Coated Lipase Derivatives

The content of PEI adsorbed on the immobilized lipase was determined by the standard 2,4,6-trinitrobenzenesulfonic acid (TNBS) assay [50]. Briefly, 0.5 g of the enzyme preparation were suspended in 5 mL of 100 mM sodium phosphate buffer (pH 8), and then 0.5 mL of TNBS commercial solution were added. After incubating the reaction mixture at 25 °C for 30 min, the colored support was exhaustively washed with 100 mM sodium phosphate buffer (pH 8). Finally, 100 mg of the treated support were suspended in 2.5 mL of sodium phosphate at pH 8 in a cuvette (1 cm path length), submitted to continuous stirring and the absorbance was recorded at 425 nm.

## 4. Conclusions

The coating of lipases immobilized on octyl supports with ion polymers strongly reduces the enzyme release during incubation under drastic conditions. The positive effect is a high stabilization of the immobilized enzyme and the prevention of the product contamination, which is very relevant in food chemistry.

This reinforcement of the adsorption may be also relevant for ion exchanged lipases, very relevant when the support utilized to immobilize the enzyme is relatively expensive like using nanoparticles [51,52,53], or other sophisticated and expensive supports [54], even immobilized lipases sued to purify or immobilize lipases may be complex to reuse after enzyme inaction [55,56]. Lipase have a broad range of applications [6,57] and in many cases the enzyme prevention of desorption but maintaining the possibility of support reuse may be a key point to utilize a support to immobilize an enzyme via a reversible adsorption like the proposed in this paper [58,59,60].

Because of this enzyme-support interaction reinforcement, it is more difficult (but still possible) to reach full enzyme desorption when it is desired to reuse the support. This side effect was expected, as the coating with ionic polymers was designed to difficult enzyme desorption via intermolecular crosslinking. Furthermore, enzyme desorption becomes much more difficult using this polymeric coated enzyme biocatalyst after submitting the enzyme to inactivating conditions. This effect is not observed when the enzyme is not coated with PEI and DS. However, untreated immobilized lipase in OC is almost fully released from octyl supports. Moreover, no PEI molecules adsorbed on octyl agarose beads remain attached to the support after heating and further incubation at high ionic strength. That is, it is necessary to have both, protein and PEI to get this adsorption reinforcement effect during incubation at high temperatures. It seems that a polymer/unfolded enzyme composite is formed during enzyme inactivation (perhaps involving several enzyme and polymer molecules). This enzyme/polymer composite has a strong capability to interact with the anionic and octyl groups that are presented in the surface of octyl-agarose supports, making the enzyme and PEI molecules desorption from the support required for its save reuse still harder. This phenomenon described here for octyl agarose may have also incidence in any hydrophobic support that presents some ionic groups on its surface. Even though those ionic groups alone have not the capability to interact with individual enzyme molecules, they can have a strong interaction with the composite formed by enzyme and ionic polymer molecules, mainly if several intermolecular crosslinked molecules are involved.

## Figures and Tables

**Table 1:** Primary amino content of polymer coated CALB and RML derivatives before and after desorption treatments. The primary amino content was determined by the TNBS assay and expressed in absorbance units at 425 nm.

Treatment	OC-CALB-PEI-DS (Without Inactivation)	OC-CALB-PEI-DS (Inactivated)	OC-RML-PEI-DS (Without Inactivation)	OC-RML-PEI-DS (Inactivated)
Without treatment	0.86 ± 0.09	0.58 ± 0.09	0.75 ± 0.07	0.50 ± 0.06
2% SDS (pH 7, 25 °C)	0.56 ± 0.07	0.51 ± 0.08	0.42 ± 0.08	0.45 ± 0.06
2% SDS in 2 M sodium phosphate (pH 7, 25 °C)	0.39 ± 0.05	0.45 ± 0.09	0.29 ± 0.05	0.38 ± 0.07
2% SDS in 2 M sodium phosphate (pH 3, 25 °C)	0.25 ± 0.06	0.32 ± 0.06	0.12 ± 0.03	0.25 ± 0.05
2% SDS in 2 M sodium phosphate (pH 3, 45 °C)	0	0.12 ± 0.04	0	0.16 ± 0.03
2% SDS in 4 M sodium phosphate (pH 2.5, 45 °C)	0	0	0	0
